# ICTV Virus Taxonomy Profile: *Chrysoviridae*

**DOI:** 10.1099/jgv.0.001383

**Published:** 2020-01-20

**Authors:** Ioly Kotta-Loizou, José R. Castón, Robert H. A. Coutts, Bradley I. Hillman, Daohong Jiang, Dae-Hyuk Kim, Hiromitsu Moriyama, Nobuhiro Suzuki, ICTV Report Consortium

**Affiliations:** ^1^​ Imperial College London, London, SW7 2AZ, UK; ^2^​ Centro Nacional Biotecnología/CSIC, 28049 Madrid, Spain; ^3^​ University of Hertfordshire, Hatfield, AL10 9AB, UK; ^4^​ Rutgers, The State University of New Jersey, New Brunswick, NJ, USA; ^5^​ Huazhong Agricultural University, Wuhan 430070, PR China; ^6^​ Chonbuk National University, Chonbuk 561-756, Republic of Korea; ^7^​ Tokyo University of Agriculture and Technology, Tokyo 183-8509, Japan; ^8^​ Okayama University, Kurashiki, Japan

**Keywords:** *Chrysoviridae*, *Alphachrysovirus*, *Betachrysovirus*, taxonomy, ICTV report

## Abstract

Members of the family *Chrysoviridae* are isometric, non-enveloped viruses with segmented, linear, dsRNA genomes. There are 3–7 genomic segments, each of which is individually encapsidated. Chrysoviruses infect fungi, plants and possibly insects, and may cause hypovirulence in their fungal hosts. Chrysoviruses have no known vectors and lack an extracellular phase to their replication cycle; they are transmitted via intracellular routes within an individual during hyphal growth, in asexual or sexual spores, or between individuals via hyphal anastomosis. This is a summary of the International Committee on Taxonomy of Viruses (ICTV) Report on the taxonomy of the family *Chrysoviridae*, which is available at ictv.global/report/chrysoviridae.

## Virion

**Fig. 1.**
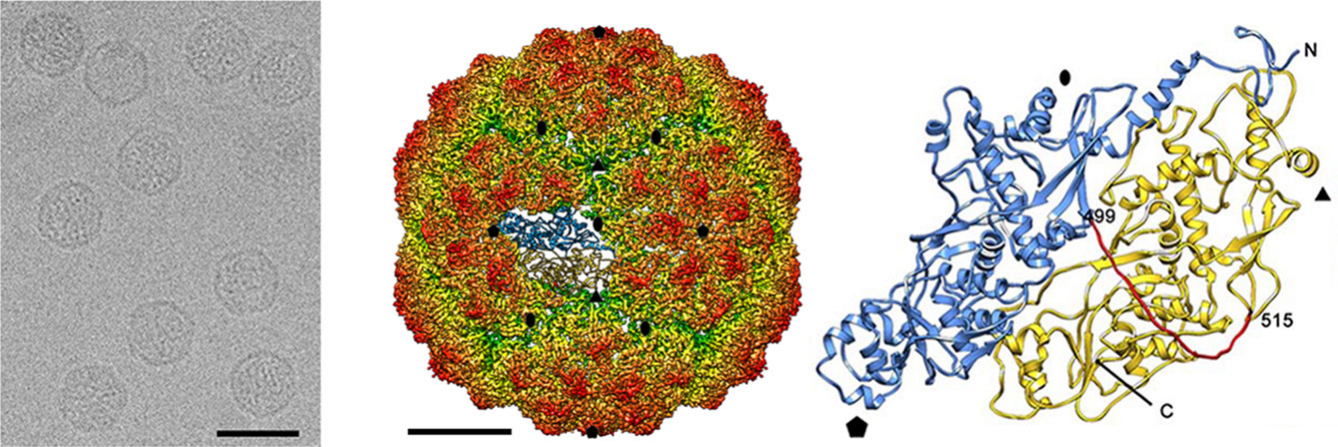
Structure of Penicillium chrysogenum virus. (Left) Cryo-EM image (bar, 50 nm), (Middle) Radially colour-coded three-dimensional cryo-electron microscopy reconstruction of the capsid viewed along a two-fold axis. The atomic structure of a monomer of the capsid protein is shown. Bar, 50 nm. (Right) Atomic model of capsid protein (top view) showing the N-terminal domain (1–498, blue), the linker segment (499–515, red) and the C-terminal domain (516–982, yellow). Symbols indicate icosahedral symmetry axes (adapted from [1]).

Virions are isometric, non-enveloped particles about 40 nm in diameter with a protein shell approximately 50 Å thick (Table 1, Fig. 1) . The Penicllium chrysogenum virus (PcV) capsid comprises 60 copies of a polypeptide arranged on a T=1 icosahedral lattice, with 12 outwardly protruding pentons each consisting of five copies of the capsid protein (CP). The CP is formed by a repeated predominantly α-helical domain, indicative of ancestral gene duplication. This basic fold is well preserved among dsRNA viruses [1].

**Table 1.** Characteristics of members of the family *Chrysoviridae*

Typical member	Penicillium chrysogenum virus ATCC 9480 (dsRNA1: AF296439; dsRNA2: AF296440; dsRNA3: AF296441; dsRNA4: AF296442), species *Penicillium chrysogenum virus*, genus *Alphachrysovirus*
**Virion**	Isometric, non-enveloped, about 40 nm in diameter
**Genome**	A total of 8.9–16.0 kbp of dsRNA in a multipartite genome (3–7 segments, usually 4) with each segment separately encapsidated
**Replication**	Particles containing both dsRNA and ssRNA can be isolated from infected fungal hosts. Virions accumulate in the cytoplasm
**Translation**	From positive-sense transcripts of genomic dsRNAs
**Host range**	Fungi, plants and possibly insects
**Taxonomy**	Realm *Riboviria*; the genera *Alphachrysovirus* and *Betachrysovirus* each include multiple species

## Genome

**Fig. 2.**
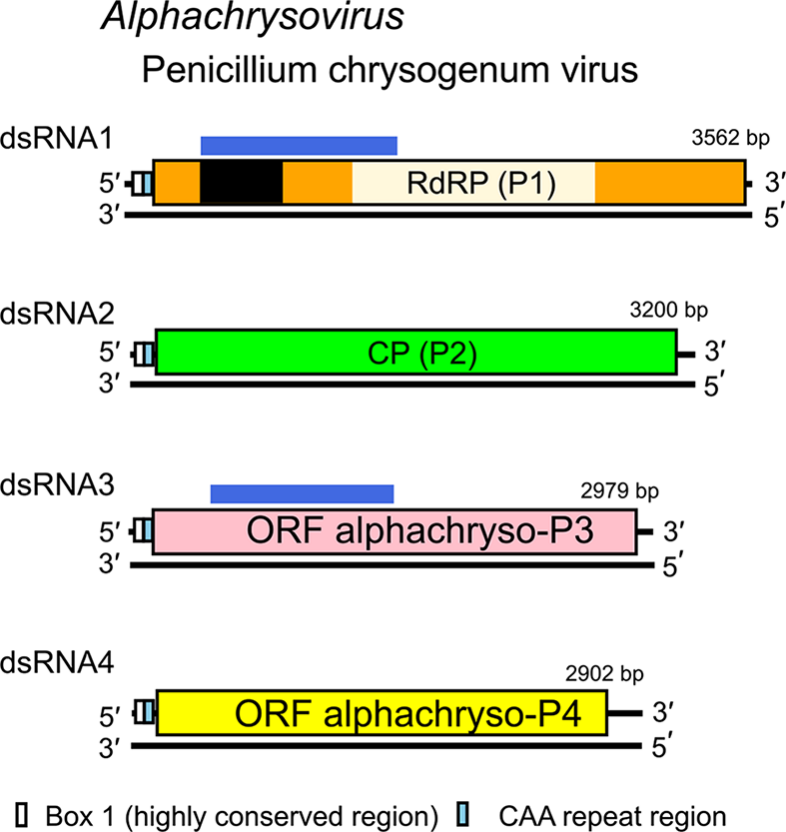
Genome organization of Penicillium chrysogenum virus. Each dsRNA segment is monocistronic. Blue bar: N-terminal region of alphachryso-P3 similar to N-terminal region of the RdRP. Alphachryso-P4 is a putative cysteine protease

Chrysovirus genomes range from 8.9 to 16.0 kbp and comprise 3–7 individually encapsidated dsRNA segments. Each segment contains a single ORF flanked by long non-coding regions (NCRs) with strictly conserved termini (Fig. 2). In addition, a 40–75 bp region with high sequence identity is present in the 5′-NCR of all four PcV dsRNAs. Immediately downstream is a stretch of 30–50 bp of CAA repeats, similar to the enhancer elements present in the 5′-NCRs of tobamoviruses.

The largest dsRNA encodes the RNA-directed RNA polymerase (RdRP) which also contains an independent P-loop NTPase domain at the N terminus. Other proteins are not conserved between members of different genera. The second largest dsRNA encodes the major capsid protein. Alphachryso-P3 has a ‘phytoreo S7 domain’, similar to that of phytoreovirus P7 proteins with nucleic acid binding activities, and its N-terminal regions share significant sequence similarity with comparable N-terminal regions of the RdRP encoded by the largest dsRNA1. Some chrysoviruses lack the dsRNA that encodes alphachryso-P3. Alphachryso-P4 contains motifs from the conserved core of the ovarian tumour gene-like superfamily of predicted cysteine proteases. Little is known about the possible functions of betachryso-P3 and betachryso-P4, or of the additional proteins encoded by chrysoviruses with more than four genomic segments.

## Replication

The virion-associated RdRP catalyses *in vitro* end-to-end conservative transcription of dsRNAs to produce mRNA. Particles containing a single molecule of dsRNA, or containing both dsRNA and ssRNA, can be isolated from an infected fungal host [2]. Virions accumulate in the cytoplasm.

## Pathogenicity

The alphachrysovirus, Aspergillus fumigatus chrysovirus, reduces growth and pathogenicity of *Aspergillus fumigatus*, the major cause of aspergillosis in immunocompromised patients [3]. The betachrysovirus Magnaporthe oryzae chrysovirus 1-A induces hypovirulence in the rice blast fungus [4], while Alternaria alternata chrysovirus 1 reduces the growth of the host fungus but renders it hypervirulent in its host plant by increasing the production of a host-specific toxin [5].

## Taxonomy

The genera *Alphachrysovirus* (previously *Chrysovirus*) and *Betachrysovirus* are distinguished by phylogenetic analysis of RdRP sequences. *Alphachrysovirus* includes viruses with three or four genomic segments, which infect fungi, plants and possibly insects. *Betachrysovirus* includes viruses with four, five and in one reported case seven [6] genomic segments, which infect both ascomycetes and basidiomycetes.

## Resources

Current ICTV Report on the family *Chrysoviridae*: ictv.global/report/chrysoviridae.
